# Interaction between autophagy and the NLRP3 inflammasome in Alzheimer’s and Parkinson’s disease

**DOI:** 10.3389/fnagi.2022.1018848

**Published:** 2022-10-03

**Authors:** Ranran Lu, Lijie Zhang, Xinling Yang

**Affiliations:** ^1^Department of Neurology, The Second Affiliated Hospital of Xinjiang Medical University, Ürümqi, China; ^2^Xinjiang Key Laboratory of Neurological Disease Research, Ürümqi, China

**Keywords:** autophagy, NLRP3, protein aggregation, inflammasome, Parkinson’s disease, Alzheimer’s disease

## Abstract

Autophagy degrades phagocytosed damaged organelles, misfolded proteins, and various pathogens through lysosomes as an essential way to maintain cellular homeostasis. Autophagy is a tightly regulated cellular self-degradation process that plays a crucial role in maintaining normal cellular function and homeostasis in the body. The NLRP3 inflammasome in neuroinflammation is a vital recognition receptor in innate cellular immunity, sensing external invading pathogens and endogenous stimuli and further triggering inflammatory responses. The NLRP3 inflammasome forms an inflammatory complex by recognizing DAMPS or PAMPS, and its activation triggers caspase-1-mediated cleavage of pro-IL-1β and pro-IL-18 to promote the inflammatory response. In recent years, it has been reported that there is a complex interaction between autophagy and neuroinflammation. Strengthening autophagy can regulate the expression of NLRP3 inflammasome to reduce neuroinflammation in neurodegenerative disease and protect neurons. However, the related mechanism is not entirely clear. The formation of protein aggregates is one of the standard features of Neurodegenerative diseases. A large number of toxic protein aggregates can induce inflammation. In theory, activation of the autophagy pathway can remove the potential toxicity of protein aggregates and delay the progression of the disease. This article aims to review recent research on the interaction of autophagy, NLRP3 inflammasome, and protein aggregates in Alzheimer’s disease (AD) and Parkinson’s disease (PD), analyze the mechanism and provide theoretical references for further research in the future.

## Introduction

Autophagy is a standard and tightly regulated cellular self-degradation process responsible for engulfing damaged organelles, misfolded proteins, and invading pathogens in a bilayer membrane called the autophagosome and guiding them for lysosomal degradation (Mizushima et al., 2008; Glick et al., 2010). Under normal conditions, excess proteins can be recycled through the autophagy-lysosomal system in the body to prevent excessive accumulation or secretion of proteins, so autophagy plays a crucial role in the metabolism and energy balance in the body (Kim and Lee, 2014; Parzych and Klionsky, 2014). Unlike other cells, neurons are non-regenerating and irreplaceable and must regulate autophagy to maintain cell survival (Muller et al., 2017; Stavoe and Holzbaur, 2019). Therefore, normal autophagy is of great significance for maintaining the survival of nervous system cells. In addition, abnormal autophagy is involved in the occurrence and progression of other diseases, such as cancer, cardiovascular disease, obesity, non-alcoholic fatty liver disease, and infection (Martinez-Lopez and Singh, 2015; Abdellatif et al., 2018; Namkoong et al., 2018; Li et al., 2020; Zhu and Liu, 2022). The inflammasome is a multi-protein signaling complex typically produced in response to stimulatory conditions by microorganisms or pathogens (Medzhitov, 2008). The NLRP3 receptor belongs to a protein family of nucleotide-binding oligomerization domain-like receptors, also known as NOD-like receptors (NLRs), which are extensively studied in the inflammasome (Swanson et al., 2019). The NLRP3 inflammasome is generally composed of three parts: the NLRP3 sensor protein [a pattern recognition receptor (PRR) that acts as a sensor molecule], the adaptor protein ASC (an apoptosis-related SPECK-like protein containing caspase activation and recruitment domains), and pro-caspase 1 (function as an effector molecule) (Jo et al., 2016; Kelley et al., 2019). Immunoreceptors of the inflammasome respond to pathogen-associated molecular patterns (PAMPs) and damage-associated molecular patterns (DAMPs) in a pattern recognition receptor (PRR)-dependent manner and subsequently mediate the activation of the inflammatory mediator caspase-1 and Induce inflammatory response, regulate the maturation and secretion of IL-1β and IL-18, and then trigger a series of inflammatory responses (Yu et al., 2014; Wang and Hauenstein, 2020). The formation of abnormal protein aggregates in neurons has been a research hotspot in neurodegenerative diseases, such as Lewy body-containing alpha-synuclein in PD, amyloid beta (Aβ) plaques in AD, and mutant huntingtin cytoplasmic inclusions in Huntington’s disease (HD), etc (Wisniewski and Konietzko, 2008; Arrasate and Finkbeiner, 2012; Rocha et al., 2018). Recent evidence suggests that microglial autophagy in the central nervous system plays an essential role in clearing abnormal protein aggregates and delaying disease progression (Su et al., 2016; Kim et al., 2017). At the same time, some studies have found that the overexpression of NLRP3 inflammasome is detected in the brains of patients with major degenerative neurological diseases, which is closely related to the occurrence and development of neurological diseases (Song et al., 2017; Milner et al., 2021). Although there is an interaction between autophagy and the NLRP3 inflammasome in microglia, and this interaction plays a vital role in many diseases, including neurodegeneration, the mechanism remains to be elucidated. This article reviews the interaction between NLRP3 inflammasome and autophagy and its mechanism of action in AD and PD to provide ideas for future related research. The current reports on the interactions between misfolded proteins, inflammatory activation signals, and autophagosome/lysosome in neurodegenerative diseases are shown in Table 1.

**TABLE 1:** Misfolded proteins, neuroinflammation, and autophagosome/lysosome interactions in neurodegenerative diseases.

Investigators	Diseases	Misfolded proteins	Inflammatory signals	Autophagosome/Lysosome
Ahmed et al., 2017	AD	Aβ, p-tau	GMF-NLRP3-Caspase-1	SQSTM1/p62
Chen et al., 2021	PD	α-syn	p38-TFEB-NLRP3	LAMP2A
Halle et al., 2008	AD	Aβ	Cathepsin B-IL-1β	LAMP1
Panicker et al., 2022	PD	a-syn	ZNF746/Paris-MitoROS	Proteasomal
Puntambekar et al., 2022	AD	Tau	CX3CR1-TGFβ	Phagocytic compartments
Ramirez-Jarquin et al., 2022	HD	mHTT	SUMO1-DARPP-32	p62, LC3B-II
Stancu et al., 2019	AD	Tau	Cathepsin-NLRP3–ASC	LAMP1
Zhou et al., 2021	AD	Aβ25–35	NLRP3-TNF-a	TFEB
Zhang et al., 2021	PD	a-sy	mGluR5-NF-κB	LAMP1
Xu et al., 2021	AD	tau	Lipid droplets-NLRP3-ROS	ATG7

AD, Alzheimer’s disease; PD, Parkinson’s disease; HD, Huntington’s disease; Aβ, amyloid beta; a-syn, alpha-synuclein; NLRP3, NOD-like receptor family pyrin domain containing 3; GMF, glia maturation factor; SUMO1, small ubiquitin-like modifier-1; TFEB, transcription factor EB; LAMP1, lysosomal-associated membrane protein 1; TNF-α, tumor necrosis factor-α; mGluR5, metabotropic glutamate receptor 5; ROS, reactive oxygen species.

## Overview of autophagy

**FIGURE 1:**
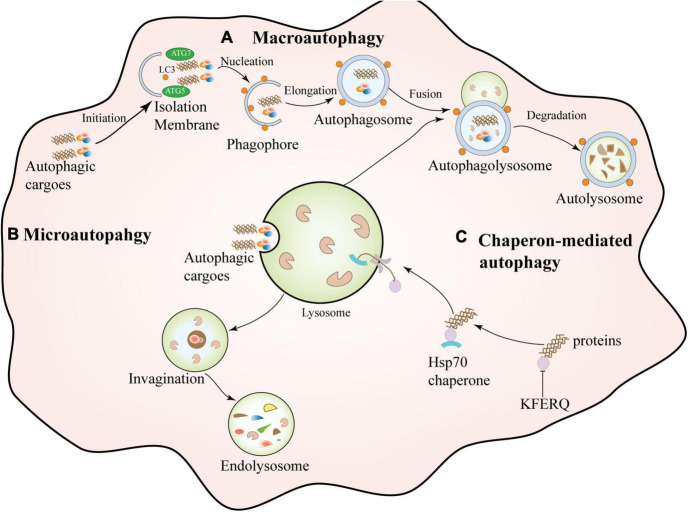
Types of autophagy. Macroautophagy is a dynamic process characterized by the sequestration of cytoplasmic contents in a bilayer membrane structure, forming an intermediate structure called an autophagosome or autophagosome and then fusion with lysosomes for degradation. Microautophagy: It encapsulates contents by invagination on the lysosomal membrane to form an intrinsic vesicle followed by degradation. Chaperone-mediated autophagy (CMA) is a highly selective catabolic process that mediates specific target motifs containing CMAs. The protein (KFERQ) is dissociated by cytosolic chaperones and transferred to the lysosome for degradation through a transmembrane complex composed of lysosome-associated transmembrane protein (LAMP2A).

Autophagy is a complex and highly conserved intracellular self-degradation process that transports aggregated or misfolded proteins, toxic cellular components, and damaged organelles to lysosomes for degradation (Glick et al., 2010; Hansen et al., 2018). As the most crucial natural self-protection mechanism, autophagy exists in almost all cells, tissues, and organs, and it mainly maintains the body’s homeostasis, nutrient metabolism, and energy balance (Mizushima, 2007; Klionsky et al., 2021). Autophagy is usually divided into different types according to the selection of different substrates and the way of transporting cargo to lysosomes, which can be divided into macroautophagy, microautophagy, and molecular chaperone-mediated autophagy (CMA) (Levine and Kroemer, 2008; He and Klionsky, 2009). Macroautophagy is the most common type of autophagy. It is a dynamic process characterized by sequestering cytoplasmic contents in a bilayer membrane structure, forming an intermediate structure called an autophagosomes or autophagic vacuoles, and then Fusion with lysosomes for degradation (Griffey and Yamamoto, 2022). Microautophagy encapsulates the contents through an invagination in the lysosomal membrane to form an internal vesicle for subsequent degradation (Schuck, 2020). The difference between microautophagy and macroautophagy is that the former degrades cytoplasmic contents through small invaginations on the lysosomal membrane without autophagosomes (Galluzzi et al., 2017). Chaperone-mediated autophagy (CMA), Unlike macroscopic and microscopic autophagy, chaperone-mediated autophagy (CMA) does not involve vesicle formation. CMA is a highly selective catabolic process by mediating proteins containing a specific target motif of CMA (KFERQ), dissociated by cytoplasmic chaperones, and composed of lysosome-associated transmembrane protein (LAMP2A) (Kaushik and Cuervo, 2018). In addition to autophagy, the ubiquitin-proteasome system (UPS) is also involved in the degradation and clearance of abnormal protein aggregates in neurodegenerative diseases (Behl et al., 2022). UPS-dependent degradation may be limited to soluble misfolded proteins or small oligomers, which are allowed to enter the P20S catalytic compartment after unfolding (Scotter et al., 2014). There is a coordinated interaction between autophagy and the UPS during protein degradation *in vivo*, mainly because when the UPS is d amaged, autophagy activation may rescue cell survival through alpha-synuclein clearance (Xilouri et al., 2013; Yuan et al., 2022). The classification of autophagy is shown in Figure 1.

### Regulation mechanism of autophagy in diseases

Autophagy is a crucial biological function for maintaining cellular homeostasis and metabolism (Klionsky et al., 2021). The study found that when autophagy changes, the abnormal accumulation of damaged organelles and abnormally folded proteins in cells can lead to irreversible damage (Linda et al., 2022). Therefore, as a critical regulator of various cellular functions, autophagy can effectively prevent the accumulation of cytotoxic products. Autophagy at the physiological level is essential for promoting cellular metabolism and responding to stress in various situations, including starvation, protein toxicity, organelle damage, and microbial infection (Levine and Kroemer, 2019; Nakatogawa, 2020).

Numerous studies have confirmed that autophagy function and pathological proteins interact in the development of neurodegenerative diseases (Ohsumi, 2014). In general, pathological protein aggregates are mainly degraded through the macroautophagy pathway, and abnormal α-synuclein or Aβ is phagocytosed by circular double-membrane phagosomes as autophagy substrates and extends and fuses to form autophagy corpuscle (Minakaki et al., 2018; Fang et al., 2019). Autophagosomes retrograde to somatic cells through the unidirectional movement of axon terminals, bind to lysosomes and unload pathological proteins to wait for complete degradation, among which the recruitment of core autophagy proteins (such as ATG5 or ATG7) plays a role in the process of autophagy essential (Zheng et al., 2019; Nishimura and Tooze, 2020). Recently, Hilverling et al. (2022) found that pH in the intracellular environment affects the transport of autophagosomes. During the fusion of autophagosomes and lysosomes, lysosomes preferentially move to the cell center, while acidic autophagosomes are transported to the periphery with high frequency. This indicates that lysosomes are first produced in the periphery, fuse with autophagosomes through transportation to the cell center, and finally undergo acidification, fusion, and transportation to the periphery (Hilverling et al., 2022). Secondly, abnormal protein aggregates can also be degraded through the microautophagy pathway, mainly mediated by cytoplasmic proteins targeted by the chaperone complex HSC70 and directly fuses into the lysosome with the invagination on the lysosomal membrane for degradation (Kaushik and Cuervo, 2018; Bourdenx et al., 2021). However, a large number of pathological protein aggregations inhibit autophagy function. For example, the accumulation of alpha-synuclein leads to the damage of autophagosome maturation and lysosome structure by including bodies containing alpha-synuclein, which ultimately reduces autophagic flux (Winslow and Rubinsztein, 2011). Recent studies have shown that alterations in autophagic flux may be related to the dependence of α-synuclein disruption on Arp2/3 actin cytoskeleton stability and intramitochondrial protein balance (Sarkar et al., 2021). However, each step in the autophagy-lysosome degradation pathway, such as vesicle transport, autophagosome formation, and fusion with lysosomes, can affect protein degradation, resulting in the accumulation of many pathological proteins in cells, thereby aggravating cell damage (Senkevich and Gan-Or, 2020; Fleming et al., 2022). Therefore, in specific pathological settings, autophagy may play a cytoprotective, survival-promoting role at early time points (Fang et al., 2019). Conversely, prolonged induction of autophagy may lead to detrimental flux dysregulation in prolonged unresolved injury, ultimately leading to apoptosis or necrosis (Di Meco et al., 2020).

In conclusion, in most autophagy lysosomal diseases, the brain is often the most severely affected organ, and neurons rely heavily on autophagy to maintain normal function and homeostasis, which indicates that autophagy plays an essential role in neuronal health. To date, autophagy dysfunction has been shown to induce neurodegeneration and exacerbate disease progression (Lee et al., 2022). However, the specific mechanisms of autophagy in neurodegenerative disease development remain unclear, as most studies use autophagy-deficient validation knockout animals or cellular models. Furthermore, validation of autophagic function in AD and PD using human tissue samples is complicated due to the limitations of methods for measuring autophagic activity. Therefore, it is necessary to explore further the regulatory role of autophagy in neurodegenerative diseases in a physiologically relevant range.

## Overview of the NLRP3 inflammasome

As a macromolecular complex composed of various proteins in the cytoplasm, the inflammasome is an essential part of the innate immune system (Guo et al., 2015). It plays a crucial role in immune protection against microbial infection. Neuroinflammation is necessary to eliminate foreign invading pathogens, clear damaged cells or abnormal proteins, and promote tissue repair in the central nervous system (CNS) (Heneka et al., 2018). However, uncontrolled neuroinflammation has been identified as a causative factor in various neurological diseases (La Vitola et al., 2021). In neurodegenerative diseases, microglia are important innate immune cells in the brain that can activate the inflammasome (Salter and Stevens, 2017; Badanjak et al., 2021). In addition, other types of CNS resident cells, including astrocytes and neurons and infiltrating monocytes from the periphery, also express and activate inflammasomes. Aβ activates the NF-κB pathway in astrocytes and leads to increased release of complement C3, which in turn acts on C3a receptors on neurons and microglia, leading to neuronal dysfunction and microglial activation (Lian et al., 2016). However, it is still controversial whether astrocyte cells can directly activate the inflammasome. Panicker et al. (2022) found that neurons might be involved in assembling the NLRP3 inflammasome. They observed that the activation of the NLRP3 inflammasome and the loss of neurons in Parkin-depleted mouse DA neurons was associated with increased Parkin substrate Paris, mitochondrial dysfunction, and the massive release of mtROS (Panicker et al., 2022). In addition, peripheral monocytes are also involved in inflammatory processes in neurodegenerative diseases. After Xu et al. (2022) used alpha-synuclein fibers to stimulate human and mouse macrophages and dendritic cells, they observed that expression of mutant LRRK2 increased the recruitment of pro-inflammatory monocytes into the brain. In addition, intravenous injection of two different recombinant alpha-synuclein pathogenic strains (fibers or bands) in wild-type mice induces an increase in the absolute number of brain-resident microglia and promotes the recruitment of peripheral blood mononuclear cells to the central nervous system (Peralta Ramos et al., 2019).

NLRP3 is the most characteristic inflammasome of the NLR receptor family and is widely expressed in immune cells (Hanslik and Ulland, 2020). It plays a role in the body’s defense against pathogen invasion. Also, it senses damaged proteins, such as misfolded or aggregated Aβ or alpha-synuclein, which may be involved in AD, PD, and other neurodegenerative diseases and neurological degeneration (Ou et al., 2021; Van Zeller et al., 2021). The NLRP3 inflammasome is generally composed of three parts: the NLRP3 sensor protein [a pattern recognition receptor (PRR) that acts as a sensor molecule], the adaptor protein ASC (an apoptosis-related SPECK-like protein containing caspase activation and recruitment domains), and procaspase-1 (function as an effector molecule) (Jo et al., 2016). The immunoreceptors of the NLRP3 inflammasome respond to PAMPs and DAMPs in a pattern recognition receptor (PRR)-dependent manner by the transcription factor nuclear factor-kappa light chain enhancer activated B cells (NF-κB) trigger the expression of pro-IL-1β and pro-IL-18 and promote the maturation and secretion of IL-1β and IL-18 by activating caspase-1 and inducing inflammation (Bauernfeind et al., 2009; Stutz et al., 2017).

### Inflammasome activation

The study found that so far, the NLRP3 inflammasome plays an inflammatory role through two steps of initiation and activation. The initiation step is induced by activating the transcription factor NF-κB by a family of pattern recognition receptors PRR proteins, such as toll-like receptor 4 (TLR4) agonists, tumor necrosis factor receptors, or ligands of the NLR family, promote NLRP3 and IL-1β and IL-18 expression (Hornung and Latz, 2010; Qiao et al., 2012). The activation step includes the recognition of the NLRP3 inflammasome agonist and the assembly and activation of the inflammasome. So far, the NLRP3 inflammasome as a response sensor can be stimulated by a variety of substances. In addition to misfolded extracellular proteins, other DAMPs can induce or aggravate neuroinflammatory responses in neurodegenerative diseases, mainly including mitochondrial dysfunction (such as the release of mtDNA, mtROS, and mtUPR), adenosine triphosphate (ATP), transcription factor A mitochondria (TFAM), and Cytochrome C (Tschopp and Schroder, 2010; Sarkar et al., 2017; Roh and Sohn, 2018; Zhong et al., 2018; de Oliveira et al., 2021). Research shows high mobility family protein 1 (HMGB1) as a typical DAMP released by necrotic or excitatory neurons. HMGB1 protein is involved in initiating and activating neuroinflammation in neurodegenerative diseases (Frank et al., 2015). It mainly exerts its biological properties by directly binding with Receptor for Advanced Glycation End Products (RAGE) and TLR4 and acts as a chemotactic or pro-inflammatory factor (Tanaka et al., 2021).

**FIGURE 2:**
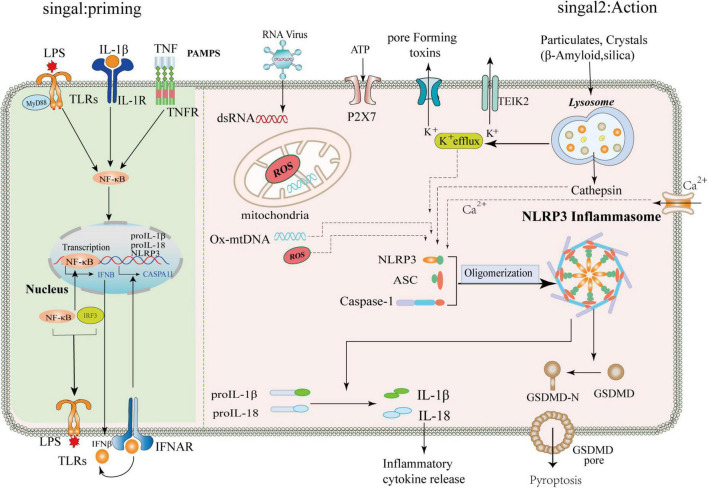
Mechanism of priming and activation of NLRP3 inflammasome. Priming signal: **(Signal 1, left)** Cells are exposed to the stimulation of various emergency factors, toll-like receptors (TLRs) and Tumor Necrosis Factor Receptor (TNFR) recognize pathogen-associated molecular patterns (PAMPS) and activate transcription factor NF-κB. NF-κB upregulates the expression of NLRP3, ProIL-1β, and pro-IL18. Activation signals: **(Signal 2, right)** The NLRP3 inflammasome can be activated by different substances, such as RNA viruses, sterile inflammation, toxic proteins, and environmental stimuli. The upstream signaling of NLRP3 is subsequently activated, which includes potassium ion (k^+^) efflux, altered calcium ion flux (Ca^2+^), mitochondrial dysfunction, and lysosomal disruption releasing cathepsins, among others. The NLRP3 protein, ASC, and pro-caspase-1 assemble into a mature complex, then convert the immature forms of IL-1β and IL-18 into mature ones. IL-1β and IL-18 are involved in subsequent inflammation, and IL-1β/IL-18 can be released into the extracellular space to propagate inflammatory signals to neighboring cells. In addition, NLRP3 activation can promote the cleavage of GSMDM by caspase-1 to form GSMDM-N and induce cell lysis.

The NLRP3 protein forms an inflammasome by processing a continuous set of signals when it experiences a specific stimulus. When extracellular fibrillar Aβ binds to TLR4 on the surface of microglia and astrocytes, it activates nuclear factor-κB and mitogen-activated protein K signaling pathways through MyD88-dependent and TRIF-dependent pathways, triggering pro-release of inflammatory factors, such as tumor necrosis factor-α, IL-1β, and IL-6 (Liu et al., 2020; Yang et al., 2020). First, NLRP3 acts as a sensor where activated and self-oligomerizes through homotypic Nacht domain interactions; oligomerized NLRP3 recruits ASCs through homotypic PYD-PYD domain interactions and induces ASCs to aggregate into a macromolecular focal point called the ASC speck (Dowds et al., 2004; Lu et al., 2014). Once NLRP3 inflammasome is activated, it induces the self-cleavage and activation of the caspase-1 (Abderrazak et al., 2015). It leads to the maturation and secretion of the pro-inflammatory cytokines IL-1β and IL-18, which may lead to a chronic inflammatory response, neuronal death, and pyroptosis of central nervous system cells (Lindberg et al., 2005). The activated downstream inflammatory factors IL-1β and IL-18 play an essential role in the nervous system (Dempsey, 2020). IL-1β can activate neuroimmune cells, activate T cells infiltrating the central nervous system, and then release IL-6 and tumor necrosis factor α, and other toxic neuromediators (Voet et al., 2019). IL-18-mediated activation of microglia enhanced caspase-1 expression, metalloproteinase levels, and pro-inflammatory cytokine production, thereby enhancing neuronal inflammation in the central nervous system. In addition, activated caspase-1 cleaves and Gasdermin D (GSDMD), which translocates to the plasma membrane and forms pores, facilitating the entry of IL-1β and IL-18 from the cytoplasm into the extracellular space (He et al., 2015). GSDMD pore formation and release of pro-inflammatory cytokines lead to a pro-inflammatory form of cell death called pyroptosis (Wang et al., 2021). Thus, the activation of the NLRP3 inflammasome is tightly regulated, and its activation is critical for host defense against pathogen invasion and maintenance of homeostasis (Shi et al., 2015). The mechanism of priming and activation of NLRP3 inflammasome is shown in Figure 2.

## Interaction between autophagy and NLRP3 inflammasome in neurodegenerative disease

**FIGURE 3:**
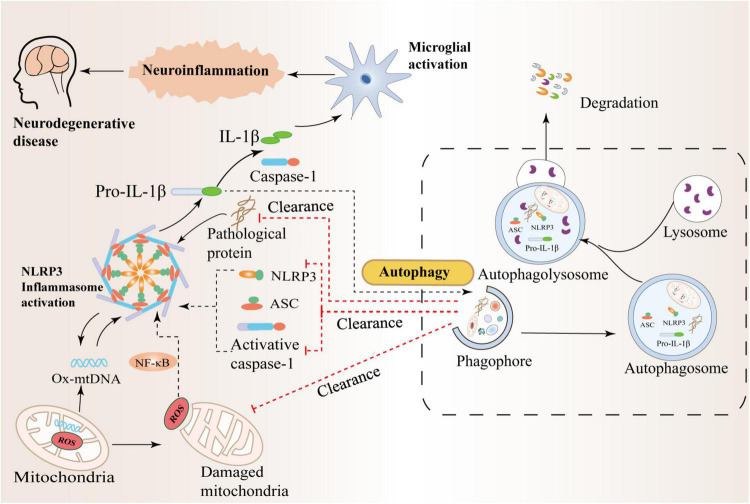
Crosstalk between autophagy and the NLRP3 inflammasome in neurodegenerative diseases. Autophagy can inhibit NLRP3 inflammasome activation by scavenging reactive oxygen species (ROS). The production of massive ROS by damaged mitochondria can activate the NF-κB pathway and promote the transcription of NLRP3 and pro-IL-1β, thereby activating the NLRP3 inflammasome. In addition, autophagy can also inhibit the activation of NLRP3 by increasing the phosphorylation of NLRP3 and degrading inflammatory components such as activated Caspase-1, IL-1β, and ASC. Therefore, there is an interaction between inflammation and autophagy to prevent excessive inflammatory responses in the body.

In recent years, studies have shown that abnormal folding and accumulation of proteins in neurons is one of the common denominators of most neurodegenerative diseases (Vaquer-Alicea and Diamond, 2019; Hulse and Bhaskar, 2022). While autophagy helps clear damaged organelles, protein aggregates, or lipid droplets, these are often unprocessed toxic substances, to a large extent, they may contribute to normal cellular dysfunction, with reduced autophagic flux further leading to autophagy deficiency or dysfunction (Alvarez-Arellano et al., 2018; Bellomo et al., 2020). Recently, a study found that α-synuclein accumulation in Lewy bodies may be due to a lack of protein clearance by chaperone-mediated autophagy and lysosomal dysfunction (Issa et al., 2018). Failure of cellular regulatory mechanisms can further reduce the rate of aberrantly aggregated proteins degraded by the proteasome and lead to massive intracellular accumulation of aberrant neurotoxic proteins, including tau and α-synuclein (Nilsson et al., 2013; Xilouri et al., 2016). High levels of IL-1β and IL-18 are present in the cerebrospinal fluid, brain tissue, and plasma of patients with central nervous system infection, brain injury, AD, and multiple sclerosis, and increased NLRP3 protein expression is associated with high IL-1β It was correlated with the serum level of IL-18, indicating that NLRP3 inflammasome activation is involved in the pathological process of neurological diseases (Karpenko et al., 2018; Irrera et al., 2020; O’Brien et al., 2020). In addition, studies have further found that IL-1β and IL-18 bind to receptors expressed on glial cells, neurons, macrophages, and endothelial cells, respectively, and initiate a series of complex signaling events that lead to inflammation in the central nervous system and intensification of the cascade reaction (Das et al., 2008; Latz et al., 2013). Therefore, the precise regulation of autophagy in neurons is significant and closely related to neuroinflammation and many aggregated proteins in brain tissue. The mechanism of crosstalk between autophagy and NLRP3 inflammasome in neurodegenerative diseases is shown in Figure 3.

### Interaction between autophagy and NLRP3 inflammasome in Alzheimer’s disease

Alzheimer’s disease is a common age-related neurodegenerative disease with an irreversible course (Knopman et al., 2021). The main clinical manifestations of AD patients are cognitive dysfunction (memory loss, visual-spatial, judgment, and decision-making deficits), progressive decline in self-care ability, and mental disorders (Serrano-Pozo et al., 2011; Long and Holtzman, 2019). The typical pathological features of AD which are senile plaques associated with the deposition of extracellular Aβ polypeptides and intracellular neurofibrillary tangles composed of hyperphosphorylated tau protein aggregates (Benilova et al., 2012; Nilsson and Saido, 2014; Tetreault et al., 2020). Studies have found that the pathogenesis of AD is related to the disturbance of Aβ homeostasis (weak clearance) and the accumulation of lysosome and its hydrolase in neurons, resulting in massive loss of hippocampal neurons, focal cortical atrophy, neuronal transsexual (Tarasoff-Conway et al., 2015).

In recent years, evidence has suggested a close link between autophagy and the deposition of protein aggregates Aβ in the pathogenesis of AD (Zhang et al., 2021). In macroautophagy, in particular, a marked accumulation of autophagosomes, immature autophagic vacuoles, and other lysosomal pre-autophagic vacuoles containing abundant Aβ deposits can be observed in the brain neurites of AD patients (Kerr et al., 2017). Studies have found that Aβ deposition may lead to impaired trafficking and maturation of autophagic vacuoles, thereby hindering the neuroprotective function of autophagy (Reddy and Oliver, 2019). However, autophagy deficiency also affects the brain’s clearance and metabolism of Aβ aggregates. Hara et al. (2006) observed significantly reduced intracellular Aβ secretion and severe neurodegeneration in autophagy-deficient APP mice. In addition, the offspring of autophagy-deficient mice exhibited a more pronounced impairment of extracellular Aβ delivery leading to the accumulation of intracellular Aβ, accompanied by memory impairment (Saito et al., 2014). Therefore, the regulatory effect between autophagy and Aβ metabolism is bidirectional, and enhancing autophagy can attenuate the excessive deposition of Aβ. Beclin1 mRNA and protein levels were detected in human and mouse AD model brain regions as an essential player in AD autophagy deficiency. However, overexpression of Beclin1 in AD mice reduced Aβ intracellular accumulation and improved neurological deficit symptoms (Bieri et al., 2018). Chronic neuroinflammation mediated by the microglia-specifically expressed NLRP3 inflammasome has been reported to play a critical role in the pathogenesis of AD (Heneka et al., 2013; Saresella et al., 2016). Saresella et al. (2016) found that the expression of NLRP3 and its related inflammatory factors in peripheral blood mononuclear cells of AD patients was positively correlated with disease severity. Terrill-Usery et al. (2014) founded the role of the NLRP3 inflammasome in the pathogenesis of AD. They found that the NLRP3 inflammasome was widely aggregated in the microglia and activated in the mouse brain stimulated by fibrillar Aβ. Activation of the NLRP3 inflammasome mediates microglia to exhibit an inflammatory M1 phenotype with high expression of caspase-1 and IL-1β (Terrill-Usery et al., 2014). In addition, activation of the NLRP3 inflammasome leads to lysosomal damage and triggers cathepsin B release, further accelerating the release of pro-inflammatory factors and chemokines, resulting in irreversible neuronal damage (Murphy et al., 2014). Recent studies have shown that the NLRP3 inflammasome is activated by fibrillar Aβ aggregates and low molecular weight Aβ oligomers and fibers. This study suggests that the central nervous system’s innate immune response triggered by Aβ activation may occur before the onset of Aβ deposition (Luciunaite et al., 2020). Heneka et al. (2013) found that deletion of NLRP3 or caspase-1 gene in APP/PS1 mice transformed microglia into the anti-inflammatory M2 type, accompanied by decreased secretion of caspase-1 and IL-1β, a significant reduction in the amount and deposition of intracellular Aβ, and a slight improvement in memory loss and behavioral abnormalities.

In AD disease progression, autophagy, NLRP3 inflammasome, and protein aggregate Aβ are closely related and have complex interactions (Hendrickx et al., 2021; Cheng et al., 2022). Ahmed et al. (2017) found that the autophagy protein SQSTM1/p62 and LC3-positive vesicles and the lysosomal marker lysosomal protein LAMP1 were increased in the temporal lobe cortex of AD patients and were associated with NLRP3 inflammasome, glial maturation factor (GMF), Aβ, and hyperphosphorylated p-tau colocalized. This may be related to the possibility that the neuroinflammation promoted by the NLRP3 inflammasome may be amplified and regulated by GMF, thereby impairing the clearance of protein aggregates mediated by the autophagosome pathway leading to impaired lysosomal integrity in AD brain temporal cortex. On the one hand, neuroinflammation can induce immune cell activation to exert neuroprotective effects. For example, in the AD cell model, Aβ induces an inflammatory response through stimulation, increasing the concentration of cellular inflammatory factors, which promotes microglia activation. Activated microglia regulate their ability to uptake, degrade and clear intracellular Aβ through phagocytosis (Cho et al., 2014). At the same time, the researchers demonstrated that NLRP3 and caspase-1 knockdown in AD mice significantly increased the ability of microglia to phagocytose amyloid Aβ and promoted the differentiation of microglia into the anti-inflammatory M2 type compared with APP/PS1 mice (Wang et al., 2017). In addition, NLRP3 or Caspase-1 inhibitors can also enhance the ability of microglia to clear Aβ, thereby reducing the accumulation of Aβ in the hippocampus of APP/PS1 mice (Dempsey et al., 2017; Alvarez-Arellano et al., 2018; Flores et al., 2020). This proves that the activation of the NLRP3/Caspase-1 inflammasome significantly reduces the phagocytosis and clearance of Aβ by microglia, thereby making it easier for Aβ to accumulate intracellularly. However, phagocytosis of excess Aβ by microglia leads to lysosomal damage in the cytoplasm and the release of cathepsin B, an endogenous danger signal for activating the NLRP3 inflammasome (Wu et al., 2017). In addition, when cells exhibit impaired or dysfunctional autophagy, Aβ degradation and clearance can be severely affected to induce the activation of the NLRP3 inflammasome. For example, after the reduction of BECN1 gene expression and the addition of autophagy blocker 3-MA, autophagy injury occurred, and the inflammatory factors IL-1β and IL-18 released by lipopolysaccharide-induced microglia were significantly higher than those in the standard group. In contrast, the expression of TNFα and IL-6 was not changed (Houtman et al., 2019). This may be related to the reduction of BECN1 affecting the processing pathways of IL-1β and IL-18. Injecting fibrillar Aβ into Atg7flox/flox/Lyz2-Cre mice increases neural tissue inflammation. This suggests that microglia degrade extracellular Aβ through autophagy and regulate the activity of the NLRP3 inflammasome (Cho et al., 2014). Researchers in a Aβ25–35 group cells and BV2 co-cultured cells added with adenovirus vector with high transcription factor EB (TFEB) expression. It was found that the expression levels of NLRP3 and other inflammatory factors in cells decreased, the level of autophagy marker LC3 decreased, and the level of lysosomal membrane protein LAMP1 increased significantly. This may be because TFEB enhanced lysosomal activity and accelerated the autophagy of lysosomes. Degradation, which ultimately facilitates the opening of autophagic flux. When the autophagic flux is unblocked, the accumulation of upstream substrates or downstream autophagic products is reduced, thereby reducing the activation of inflammatory cells by toxic substances (Zhou et al., 2021). In conclusion, enhancing microglial autophagy and inhibiting NLRP3 inflammasome activation may be a new strategy for treating AD.

### Interaction between autophagy and NLRP3 inflammasome in Parkinson’s disease

Parkinson’s disease is a common chronic, progressive degenerative disease of the central nervous system (Dorsey et al., 2018). The clinical manifestations of PD patients include movement disorders (resting tremor, bradykinesia, rigidity, and postural instability) and non-movement disorders (hyposmia, cognitive impairment, and sleep disturbance) (Jankovic, 2008; Bloem et al., 2021). The pathological hallmarks of PD are the loss of dopaminergic (DA) neurons in the substantia nigra pars compacta and the formation of pathologically misfolded protein aggregates (Cacabelos, 2017). Aggregates of misfolded alpha-synuclein (called Lewy bodies) and dysfunctional cellular debris have been found in animal models of PD to trigger a cascade of immune defenses that lead cells to produce large amounts of cytokines and other inflammatory factors and cause irreparable neuronal damage (Atik et al., 2016; Rocha et al., 2018).

Studies showing that SNCA is degraded by macrophages and CMA in neuronal cells again demonstrate the importance of autophagy processes as degradation mechanisms in the central nervous system and when that damage to these systems, especially CMA, leads to the accumulation of neurotoxic SNCA aggregates (Ho et al., 2020). Autophagy-activating Beclin-1 gene transfer also ameliorated pathological changes in limbic system synapses and dendrites and reduced SNCA accumulation in PD patients (Spencer et al., 2009). Tu et al. (2021) found that extracellular α-synuclein can inhibit autophagy initiation in microglial cells. Autophagy damage in cytoplasmic cells disrupts the autophagic activity of microglia, thereby synergistically promoting the development of neuroinflammation and Parkinson’s disease. In addition, lysozyme 2Cre (Lyz2cre)-mediated deletion of microglial autophagy-related gene 5 (ATG5) aggravates neuroinflammation and loss of dopaminergic neurons in the substantia nigra and aggravates the loss of α-synuclein overexpressing mice (Tu et al., 2021). In addition, α-Syn leads to microglial activation by activating TLR4 and its downstream p38 and Akt-mTOR signaling (Chen et al., 2021). Miki et al. (2018) found that the autophagy core regulator genes ULK3, Atg2A, Atg4B, Atg5, Atg16L1, and histone deacetylase six mRNAs were downregulated when they studied the peripheral blood mononuclear and cell-based autophagy of Parkinson’s patients, and the autophagy protein ULK1 was downregulated, Beclin1 protein levels were significantly increased, and the mRNA expression of these proteins was negative feedback and correlated with increased α-synuclein levels. In recent years, it has been reported that the inflammasome NLRP3 is involved in the pathological process of Parkinson’s disease. Recent studies have found that the gene expression of NLRP3, ASC, and caspase-1 is increased in peripheral blood mononuclear cells of PD patients, and increased protein levels of NLRP3, caspase-1, and IL-1β. In contrast, the plasma IL-1β level was significantly higher than that of the standard control group (Fan et al., 2020). von Herrmann et al. (2018) found in the histological sections of the midbrain of PD patients that DA neurons in the tissues were significantly less than those in the healthy control group, and CASP1 immunoreactivity was increased. The NLRP3 mRNA and protein levels in the midbrain homogenate of PD patients increased, confirming that DA neurons were a potential cellular source of PD inflammasome activity (von Herrmann et al., 2018). It has been found that in MPTP-treated PD mouse models, when NLRP3 deficiency can alleviate motor dysfunction and microglia-mediated activation and release of inflammatory factors in mice, thereby alleviating neuronal apoptosis (Lee et al., 2019).

There are complex interactions between autophagy defects and NLRP3 inflammasome activation in PD. Qin et al. (2021) found that deletion of Atg5 in microglia exacerbated NLRP3 inflammasome activation, dopaminergic neurodegeneration, and mouse motor dysfunction in acute and subacute MPTP mouse models, with concomitant, there is microglia and astrogliosis. At the same time, it was verified *in vitro* experiments that the inflammation intensified after the autophagy destruction of BV2 cells, and the inhibition of microglia autophagy was harmful to the cultured neurons (Qin et al., 2021). Inhibition of the NLRP3 inflammasome by constructing NLRP3 knockout mice not only prevented substantia nigra dopaminergic degeneration and striatal dopamine loss in PD mice but also prevented the formation of pathological α-synuclein in the substantia nigra (Huang et al., 2021). Furthermore, it inhibited MPTP-induced midbrain glial responses in mice while secreting pro-inflammatory cytokines. Most importantly, it alleviates autophagy dysfunction in the midbrain of PD mice (Gordon et al., 2018). Improved autophagy function involves the preventive effect of LRP3 inflammasome inhibition on α-synuclein pathology in Parkinson’s disease. Finally, this persistent autophagy dysfunction may release activated intracellular lysosomal enzymes and lead to cell death (Gordon, 2018). Thus basal levels of autophagy are important for the clearance of protein aggregates, and the reduction of cytoplasmic inclusions may have a protective effect on neurodegenerative diseases. These results suggest that α-synuclein can provide the initiation signal for the activation of the NLRP3 inflammasome. However, numerous studies in cell experiments and animal models have confirmed that activation of the NLRP3 inflammasome can also, in turn, lead to increased α-synuclein deposition and diffusion in glial cells, thereby inducing α-synuclein into a positive feedback loop and ultimately promoting PD disease progression.

## Conclusion and future perspectives

In summary, autophagy is an important mechanism affecting neuronal health, which maintains normal neuronal function by regulating lysosomal function to clear abnormally folded proteins in the nervous system. However, when autophagy is impaired, or autophagic flux is reduced, the incomplete clearance of protein aggregates can lead to mitochondrial damage, ROS generation, lysosomal disruption, and tissue protein release *in vivo*. On the other hand, many protein aggregates and intracellular release factors can further activate the NLRP3 inflammasome to induce neuroinflammation. Neuroinflammation is a double-edged sword that acts as a defense mechanism during acute infection and has anti-infective effects. However, after entering the chronic inflammatory phase, excessive release of cytotoxic factors leads to inflammatory activation that exacerbates cellular damage and neurodegeneration. Thus, hyperactivated NLRP3 inflammasome, in turn, exacerbates pathology and accelerates neurogenic disease progression. Therefore, in the early stage of the disease, we can use autophagy-enhancing drugs combined with inflammasome inhibitors to clear the aggregation of abnormal proteins and release cellular inflammatory factors, thereby improving neurodegeneration caused by cell damage and delaying disease progression.

## Author contributions

RL and LZ were the major contributors in designing the review and writing the manuscript. XY revised the manuscript. All authors contributed to the article and approved the submitted version.
